# 917. Accessible HCV Care for People Who Inject Drugs: Randomized Clinical Trial Comparing Low-Threshold Treatment at a Syringe Service Program Versus Facilitated Referral

**DOI:** 10.1093/ofid/ofab466.1112

**Published:** 2021-12-04

**Authors:** Benjamin Eckhardt, Yesenia Aponte-Melendez, Chunki Fong, Shashi Kapadia, La Davis, Melinda Smith, Pedro Mateu-Gelabert, Kristen Marks, Kristen Marks

**Affiliations:** 1 NYU School of Medicine/Bellevue Hospital, New York, NY; 2 CUNY School of Public Health, New York, New York; 3 Weill Cornell Medicine, New York, NY

## Abstract

**Background:**

To achieve hepatitis C virus (HCV) elimination, treatment programs need to be developed to engage, treat, and cure people who inject drugs (PWID).

**Methods:**

We present final data from the Accessible Care Trial for curing HCV in PWID. The randomized clinical trial compared on-site, low-threshold HCV treatment with care-coordination at a NYC syringe service program (*Accessible Care*) with facilitated referral to local providers through a patient navigation program (*Usual Care*). Eligible participants were HCV RNA+ and had injected drugs in the past 90 days. Participants were randomized 1:1 to the Accessible Care or Usual Care arm. The primary endpoint was achievement of sustained virologic response (SVR12) within 12 months of enrollment. Secondary endpoints examined rates HCV care cascade step completion from referral to clinician, attending clinical visit, baseline lab testing, treatment initiation, and cure.

**Results:**

Among the 572 participants screened, 167 met eligibility criteria and were enrolled, with two excluded post-randomization (N=165). Demographics were similar with a median age of 41 years; 22% women; 59% Hispanic and 5% non-Hispanic black. At baseline, 84% of participants had injected drugs in the past 30 days with those averaging 22 injections/month, 70% were receiving either methadone or buprenorphine, 57% were recently homeless, 7% were HIV+, and 11% were HCV treatment experienced. In the intention-to-treat analysis, 55/82 (67.1%) of the Accessible Care arm and 19/83 (22.9%) of the Usual Care arm achieved SVR12 (p< 0.001). The SVR12 rates of those starting therapy were 55/64 (85.9%) and 19/22 (86.3%) in the two arms (p=0.96). Loss to follow-up (12.2% and 16.9%, p=0.51) was similar in the two arms. Significantly more participants in the Accessible Care arm achieved all steps in the care cascade with the greatest attrition in the Usual Care arm seen in referral to clinician and attending clinical visit.

**Conclusion:**

Among HCV RNA+ PWID significantly higher rates of cure were achieved using the Accessible Care model focused on low-threshold, destigmatized, flexible HCV care compared to facilitated referral. To achieve HCV elimination, expansion of treatment programs specifically geared toward engaging PWID is paramount.

**Disclosures:**

**Benjamin Eckhardt, MD, MS**, **Gilead Sciences** (Grant/Research Support) **Shashi Kapadia, MD**, **Gilead Sciences Inc** (Grant/Research Support) **Kristen Marks, MD**, **Gilead Sciences** (Grant/Research Support)

